# Accelerating Information Retrieval from Profile Hidden Markov Model Databases

**DOI:** 10.1371/journal.pone.0166358

**Published:** 2016-11-22

**Authors:** Ahmad Tamimi, Yaqoub Ashhab, Hashem Tamimi

**Affiliations:** 1 College of Information Technology and Computer Engineering, Palestine Polytechnic University, Hebron, Palestine; 2 Palestine-Korea Biotechnology Center, Palestine Polytechnic University, Hebron, Palestine; Texas A&M University College Station, UNITED STATES

## Abstract

Profile Hidden Markov Model (Profile-HMM) is an efficient statistical approach to represent protein families. Currently, several databases maintain valuable protein sequence information as profile-HMMs. There is an increasing interest to improve the efficiency of searching Profile-HMM databases to detect sequence-profile or profile-profile homology. However, most efforts to enhance searching efficiency have been focusing on improving the alignment algorithms. Although the performance of these algorithms is fairly acceptable, the growing size of these databases, as well as the increasing demand for using batch query searching approach, are strong motivations that call for further enhancement of information retrieval from profile-HMM databases. This work presents a heuristic method to accelerate the current profile-HMM homology searching approaches. The method works by cluster-based remodeling of the database to reduce the search space, rather than focusing on the alignment algorithms. Using different clustering techniques, 4284 TIGRFAMs profiles were clustered based on their similarities. A representative for each cluster was assigned. To enhance sensitivity, we proposed an extended step that allows overlapping among clusters. A validation benchmark of 6000 randomly selected protein sequences was used to query the clustered profiles. To evaluate the efficiency of our approach, speed and recall values were measured and compared with the sequential search approach. Using hierarchical, *k*-means, and connected component clustering techniques followed by the extended overlapping step, we obtained an average reduction in time of 41%, and an average recall of 96%. Our results demonstrate that representation of profile-HMMs using a clustering-based approach can significantly accelerate data retrieval from profile-HMM databases.

## Introduction

With the exponential growth of biological sequence data, there has been an increasing interest in classifying records that share similarities in sequences, functions and structures together into families [1]. The representation of the protein, DNA, or RNA sequences into families has proven very useful to gain insight into the biological functions, molecular mechanisms, and evolutionary relationships of these biological molecules. Furthermore, detection of remote homology is more sensitive when a sequence query is searched against a family database than a sequence database [2]. Several approaches, such as regular expression, position weight matrix, and profiles, have been used to represent families of related biological sequences [3]. Currently, Profile Hidden Markov Model (profile-HMM) is one of the most sensitive approaches that has been used to represent similar biological strings as families [4]. The hallmark of profile-HMM is its ability to statistically represent the subtle conserved features of a given family of sequences.

Among the different biological data, protein sequences have received the most attention. Protein families are commonly represented and analyzed using profile-HMM. Currently, there are several protein family databases that utilize profile-HMM representation including Pfam, TIGRFAM, SUPERFAMILY, and CATH/Gene3D [5–8]. As more and more high-throughput sequence data are produced from a wide range of organisms, the contents of these databases are increasing enormously. For example, Pfam database which was launched in 1996 with 100 families that were built from 10431 sequences has in its 30^*th*^ release 16306 family profiles that were built from 12845974 sequences [5].

The advantage of creating profile-HMMs that represent homologous proteins, domains, and motifs, is the ability to perform a much more sensitive database search than the conventional pairwise sequence searching methods such as BLAST [9] and FASTA [10]. This superior feature of profile-HMMs has motivated several research groups to develop algorithms to solve sequence-profile and profile-profile comparison problems. SAM [11, 12], Prof_Sim [13], COMPASS [14, 15], HHsearch [2], PRC [16], HMMER [17], and AlignHUSH [18], are among these algorithms. The search is typically carried using pairwise sequence-profile or profile-profile alignment methods. Where, each query is always searched in a pairwise alignment manner (sequential search), i.e. the query is compared with every single profile-HMM in the target database.

The accuracy value of these methods has been enhanced by incorporating structural, physicochemical and evolutionary informative features that can help in better aligning the functionally related residues [19]. For example, HHpred [20] and the freely available HHsearch, which were developed by the group of Johannes Söing, annotate the profile-HMMs with predicted secondary structure features in order to enhance the homology detection sensitivity [20].

Despite improved sensitivity over the conventional pairwise sequence alignment, the major limitation of exploiting profile-HMM in homology search is the speed factor. Several research groups have attempted to solve this problem. In 2005, Johannes Söding increased the speed in the HH-SUITE [2] by proposing some simplifications to the alignment algorithm that excluded specific states and limited the transition between specific pair states. In 2011, Sean R. Eddy presented an acceleration heuristic for profile-HMMs for the HMMER3 tool by proposing “multiple segment Viterbi” algorithm which led to a significant increase in the searching speed of HMMER3 compared to the previous version HMMER2 [17].

The proposed solutions mainly focused on improving the speed of the pairwise alignment algorithm while maintaining the sequential search. In this work, we propose an additional speed enhancement by reducing the search scope through clustering the profile-HMM database. The approach of clustering records of biological databases to improve searching efficiency has been applied for sequence databases [21–23]. However, to the best of our knowledge, our work is the first attempt to cluster Profile-HMM data to accelerate information retrieval, especially in the case of batch querying.

The proposed technique works by initially clustering the profile-HMMs into a certain number of clusters. The distance between the profiles is measured through HHsearch homology score [2]. Then a representative is assigned to each cluster. For retrieval, a query profile-HMM is compared with the set of representatives. The cluster corresponding to the representative most similar to the query is searched for the target profiles. The proposed enhancement can be combined with different searching algorithms to increase speed and save several minutes to hours for large batch queries, with an acceptable cost on recall value.

## 1 Materials and Methods

The profile-HMMs of the TIGRFAMs database [6] were used to demonstrate the proof-of-concept of our proposed approach. We used TIGRFAMs release 13.0 with 4284 families. The 4284 profiles represent a total of 55503 protein sequences. For each protein family, TIGRFAMs has three components. The first component is the set of protein sequences that belongs to the family. These proteins are stored as multiple sequence alignments (MSA) and are usually referred to as seed sequences. The second component is the profile-HMM for the MSA of the first component. The third component is the associated information designed to support the automated functional identification of proteins by sequence homology.

HHsearch tool [2] was used for building profile-HMMs from the input MSA using *hhmake* script. To create profile-HMM databases for the representatives of each cluster data set, we used *hhblitsdb.pl* script. The script *hhsearch* was used to search and retrieve matches from the created profile-HMMs databases for a given input query.

Protein sequence data from the UniRef50 data set [24] were used for parameter tuning and validation. UniRef50 is one of the protein cluster data sets that are available through Uniprot database [25]. The seed sequences of each UniRef50 cluster have at least 50% sequence identity and 80% overlap with the longest sequence in the cluster. For parameter tuning purposes we randomly selected 100 sequences from the UniRef50 data set. However, for final validation, we randomly selected 6000 sequences.

Implementation was carried out using MATLAB [26] in addition to shell scripting on CentOS Linux operating system. Shell scripting was used in order to effectively utilize the number of cores in the server in a multi-threaded manner. The server has 32 cores (2.2 GHz) with 128G as a total RAM size.

The testing phase was carried out using a single core computer in order to study the performance of the proposed approach when running it on low computational power servers.

## 2 Algorithms

In this section, we present two approaches for building the retrieval system based on the both ‘crisp’ and ‘overlap’ clustering.

### 2.1 Crisp clustering approach

This approach consists of four steps. A detailed explanation of each step is discussed below:

#### Retrieval of protein family information

In this step we retrieve all the sequence seeds that are used to build the profiles in TIGRFAMs database. We use these seeds to create a Profile-HMMs using *hhmake* script in HH-SUITE [2]. The reason to create profiles using HH-SUITE instead of using the TIGRFAMs is to increase the homology detection sensitivity as recommended by Söding [2] in the HH-SUITE manual.

#### Build a similarity matrix

Assume Λ = [λ_1_, λ_2_, …, λ_*N*_] is a database of *N* profile-HMMs. An iterative process is used to compare each profile λ_*i*_ against all profiles in Λ. The local alignment option of HHsearch was used as it can produce more similarity results and hence increases the chance of generating clusters of profiles. This comparison process produces an output with *N* scores. The scores Each represents the profile-profile comparison score between λ_*i*_ and each profile in Λ. These scores are identified based on a measure that was developed by Söding, which was called *probability*. This measure is calculated not only based on the resulted E value of the search, but it also takes into account the secondary structure similarity. According to Söding, using the probability measure is more sensitive to identify homology. The *probability* can range from 0 to 100% and when it is larger than 95%, the homology is nearly certain [2]. We adopted this score as the similarity score throughout this paper.

These scores are used to produce an all-against-all similarity matrix *S*. The matrix *S* has *N* × *N* elements, where the element *s*_*ij*_ = *score*(λ_*i*_, λ_*j*_) represents the similarity score between query λ_*i*_ and template λ_*j*_. It is important to notice that *score*(λ_*i*_, λ_*j*_) ≠ *score*(λ_*j*_, λ_*i*_), *i* ≠ *j*, because the score depends on the length of the query. As *S* is an a symmetrical matrix, we decided to use the approach in [27] by only storing the maximum score of *s*_*ij*_ and *s*_*ji*_.

#### Clustering

At this point, the matrix *S* is used as input for the clustering process. *S* is considered as a data set of *N* samples, each sample has *N* dimensions.

Three different clustering algorithms, *k*-means, hierarchical, and connected component were used.

#### Assignment of representative profile

A representative *γ*_*i*_ for the cluster *c*_*i*_ is selected using two alternatives. The first method (which we denote as **MSA-representative**) builds the representative by retrieving all profile seeds in *c*_*i*_ and creating their multiple sequence alignment (MSA) before creating a profile-HMM based on this MSA. The second method (which we denote as **heuristic-representative**) is to select a profile λ_*i*_ from *c*_*j*_ as a representative for cluster *j* using a heuristic function *f*. This function finds the profile in the cluster that has the highest homology to all other profiles as follows:

Given a cluster of size *T*. The similarities among its profiles are in *S*′. Where *S*′ is a subset of *S*. The function *f*(λ_*i*_) returns a value that represents the likeliness of having λ_*i*_, as heuristic-representative.

$$\begin{array}{c} f\left(\lambda_{i}\right)=\sum_{j=1}^{T}S^{\prime }\left(i,j\right) \end{array}$$ (1)

The heuristic-representative is calculated by selecting the profile with the maximum similarity summation among all profiles in the cluster (i.e. *argmax*(*f*(λ_*i*_))).

**Fig 1:**
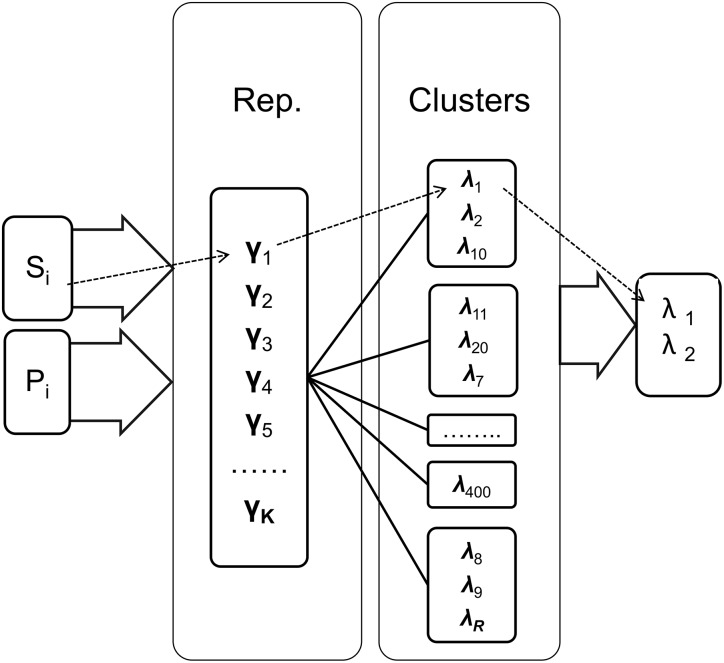
Block diagram of the retrieval technique: The dashed arrows represent an example for searching for the sequence *S*_*i*_ (or its corresponding profile *P*_*i*_). *S*_*i*_ is matched with the representative *γ*_1_. *γ*_1_ belongs to a cluster that has three profiles: λ_1_, λ_2_ and λ_10_. Finally the matched outputs are λ_1_, λ_2_.

Fig 1 shows the diagram of the proposed technique. The system first aligns the input query λ_*q*_ to Γ, which is a data set of the Representative profiles to obtain the best match. Then, the system performs a search inside the cluster from which the best matching profile originated in order to find similar profiles. The final output is a list of similar profiles in the database that matches the input query using local alignment with a similarity score greater than a threshold *ϕ*.

### 2.2 Overlapping clustering approach

In order to enhance the sensitivity of searching a further step was introduced to the four steps mentioned above. The enhancement was achieved by proposing an extended step that allows overlapping among clusters. The overlapping was made by assigning a representative for each cluster as in Section 2.1, then re-assigning the profiles in the database to clusters as follows:

Suppose that *C* = [*c*_1_, *c*_2_, …, *c*_*k*_] are the *k* clusters that are produced after clustering Λ with any of the three clustering algorithms, and Γ = [*γ*_1_, *γ*_2_, …, *γ*_*k*_] are the *k* representatives for clusters in *C*.

Then, each profile λ_*j*_ is assigned to cluster *c*_*i*_ in *C* as Eq (2) shows.

$$\begin{array}{c} c_{i}\leftarrow c_{i}\cup \lambda_{j}⟺score\left(\gamma_{i},\lambda_{j}\right)\geq \vartheta ,\;j=1,2,\ldots ,N \end{array}$$ (2)

where *ϑ* is a self-regulated threshold that is calculated as in Eq (3). This threshold is computed as the mean value of similarity vector between λ_*j*_ and profiles in Γ. The self-regulated threshold guarantees that each profile is assigned to at least one cluster.

$$\begin{array}{c} \vartheta =\frac{1}{K}\sum_{i=1}^{K}score\left(\gamma_{i},\lambda_{j}\right) \end{array}$$ (3)

The above is one heuristic way to select the threshold for the overlapping. Nevertheless, this opens other ideas that can be researched in the future.

Although sequential techniques are suppose to find the most similar profiles, they are slow since they perform *N* comparison operations for a given search in a database of *N* profiles. On the other hand, indexing speeds up the search time but may cause the query profile to be mismatched with the correct cluster.

Three scoring stages exist in our system. The first one is when comparing the query with the representatives in Γ, which costs *K* comparison operations. In the second stage, for the best representative match *γ*_*i*_, the technique starts a sequential search inside *c*_*i*_ cluster to find the matches for the input query. In the final stage, a process of sequential searching is performed within *singleton clusters* [28] (clusters having one profile). For an singletons, the technique will perform an additional *M* comparisons. The total number of comparisons needed for retrieving a query is denoted by *NC* as shown in Eq (4).

$$\begin{array}{c} NC=K+size(c_{i})+M \end{array}$$ (4)

where *c*_*i*_ is the cluster of the most similar profile to the query.

In Eq (4), the ideal case will be achieved if all clusters have the same size and *M* = 0. On the other hand, the worst case is when the clustering algorithm produces only one cluster which eventually exceeds the sequential search due to the indexing overhead. In this work, we tune the parameters of the clustering approaches empirically to have a balance between the number of clusters *K* and the size of each cluster as shown in section 3.1. It is important to note that our proposed overlapping extension step guarantees that an *M* = 0 case is achieved.

### 2.3 Evaluation criteria

The evaluation of the proposed retrieval technique was performed based on two criteria. The first one is the *reduction in time (RT)*, which is the ratio of the time to accomplish retrieving a query from *S* in comparison to the sequential search time, subtracted from 1 as shown in Eq (5).

$$\begin{array}{c} RT=1-\frac{\sum_{i=1}^{T}\alpha \left(s_{i}\right)}{\sum_{i=1}^{T}\alpha^{\prime }\left(s_{i}\right)} \end{array}$$ (5)

where *S* = [*s*_1_, *s*_2_, …, *s*_*T*_] is a query set of size *T*, *α* is the search time using the proposed technique and *α*′ is the search time using sequential technique. The second criterion is *Recall*, which is the ratio of the number of relevant matches retrieved by the proposed technique to the total numbers of relevant matches,

$$\begin{array}{c} Recall=\frac{|\beta^{\prime }\left(s_{i}\right)⋂\beta \left(s_{i}\right)|}{|\beta^{\prime }\left(s_{i}\right)|} \end{array}$$ (6)

where *β* is the set of matches using the proposed technique and *β*′ is the set of matches using the sequential technique.

In information retrieval contexts, *Recall* is usually accompanied with *Precision*, which detects the number of irrelevant matches.

In the case of information retrieval from Profile-HMM Databases, only highly similar matches are considered as homologous matches. For example, using local alignment in the sequential approach in [2], a similarity scores that are above or equal to 95% are considered as homologous matches. We follow this notion in the current research and we assume that the sequential retrieval results are all relevant. The set of retrieved matches in the our proposed method will always be a subset of the matches in the sequential technique. Consequently, Precision is not taken into consideration in this research.

To generalize the concept for calculating *Recall* for each experiment, we formulate Eq (7), which reflects the ratio between the total number of matches found by the retrieval technique to the total number of matches found using the sequential search.

$$\begin{array}{c} Recall=\frac{\sum_{i=1}^{T}\beta \left(s_{i}\right)}{\sum_{i=1}^{T}\beta^{\prime }\left(s_{i}\right)} \end{array}$$ (7)

The reduction in time and recall ratio are measured during the retrieval process of any input query. We have not taken into account the time for establishing the clusters nor the time for establishing the representatives in our experiments. This is because the establishment process is done only once. It is still worth mentioning that the establishment process is time consuming. For example, in the case of *k*-mean clustering, more than 300 hours were required to build the MSA-representative when *k* = 160.

## 3 Results and Discussion

Experiments were carried out to study the following: The performance of the three clustering algorithms; approaches for assigning representative, and the effect of introducing overlapping.

### 3.1 Parameter tuning

In order to select the best values for the clustering parameters *number of clusters* (*K*), *cutoff the hierarchical tree* (*ζ*), and *edge weight* (*ψ*), we used an independent data set of protein sequences. These sequences were randomly retrieved from UinRef50 in order to minimize any possible bias towards data from our profile-HMM TIGRFAM database. In the experimental details section, we provided detailed results of different experiments that aimed at tuning clustering parameters. The selected values of the three parameters are shown in Table 1. It is important to underscore that the obtained parameters are specific to TIGRFAM release 13, which was used for validating our approach. Therefore, a different database would require re-tuning these parameters.

**Table 1:** Parameter values after tuning.

Clustering approach	Parameter	Best value
*k*-means	*k*	160
hierarchical	*ζ*	0.9
connected component	*ψ*	95%

### 3.2 Assignment of representatives

During the tuning of the parameters of each clustering approach, we assigned representatives for each cluster using heuristic-representative approach. In this part of the experiment, we studied the other alternative of selecting the representative which is MSA-representative.

Six different comparisons were performed to cover all combinations of having three clustering algorithms and the two methods for choosing the representatives. We used the same parameter values as in Table 1

**Fig 2:**
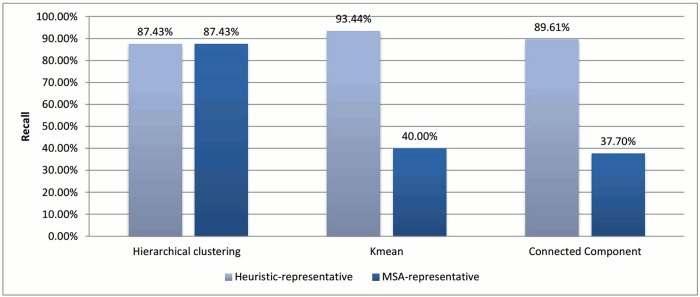
Recall comparisons for different assigning representative methods.

**Fig 3:**
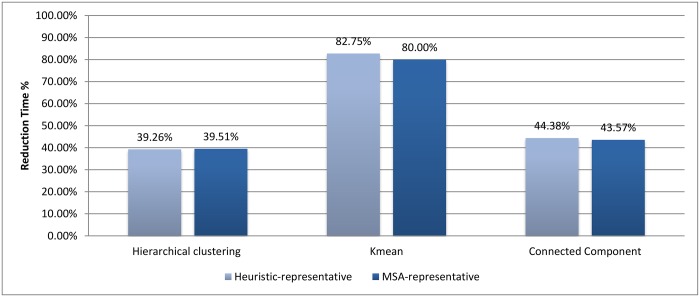
Reduction in time comparison for different assigning representative methods.

Figs 2 and 3 show the evaluation for each clustering algorithm using the two methods of assigning representatives.

**Fig 4:**
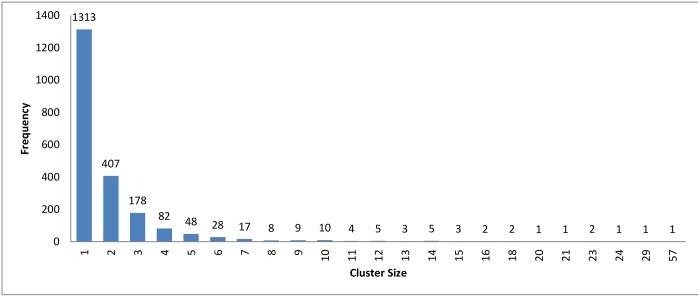
Hierarchical clustering: Clusters distribution for cutoff (*ζ*) = 0.9.

**Fig 5:**
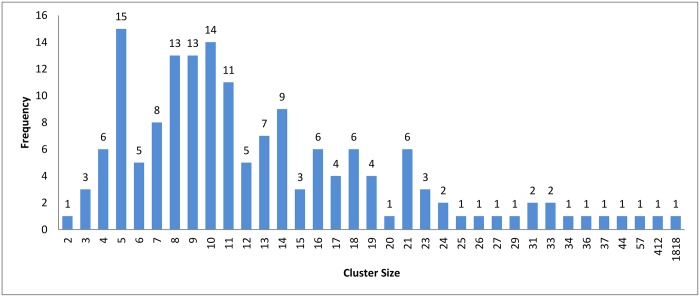
*k*-means clustering: Cluster distribution for *k* = 160.

**Fig 6:**
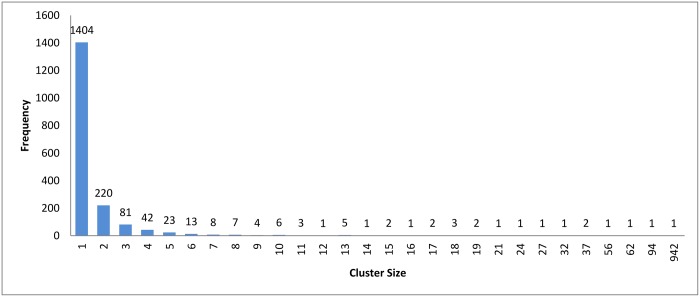
Connected Component clustering: Clusters distribution for threshold (*ψ*) = 95%.

In the case of *k*-means and connected component algorithms, better recall values were reached when a heuristic-representative was used compared to MSA-representative. There was no significant difference in recall value between heuristic-representative and MSA-representative when using the hierarchical clustering algorithm. These results are consistent with the cluster distributions for each approach in Figs 4–6. The maximum cluster size in the hierarchical algorithm was 57, while it equals 1818 and 942 for *k*-means and connected component, respectively.

As for reduction in time, both ways of selecting the representative lead to a similar reduction in time. So, the method of selecting the representative has a greater effect on accuracy than on time reduction.

### 3.3 Application of overlapping

In *crisp* clustering techniques, each item is assigned to only one of the existing clusters. It is possible to have items shared between more than one cluster by introducing overlapping. This is suitable in our case because we employ local alignment, where one or more local segments from the same profile can be similar to different profiles segments.

However, the profile similarity is not transitive through the scoring process that was used. This means if *score*(λ_*a*_, λ_*b*_) ≥ *ϕ*, and *score*(λ_*b*_, λ_*c*_) ≥ *ϕ* under a given threshold *ϕ*, it does not necessarily mean that *score*(λ_*a*_, λ_*c*_) ≥ *ϕ*.

If we use crisp clustering, λ_*a*_, λ_*c*_ may be assigned to two different clusters and λ_*b*_ is assigned to one of them. However, λ_*b*_ should be assigned to both clusters at the same time because it is similar to both of them. This is handled by the overlapping technique.

**Fig 7:**
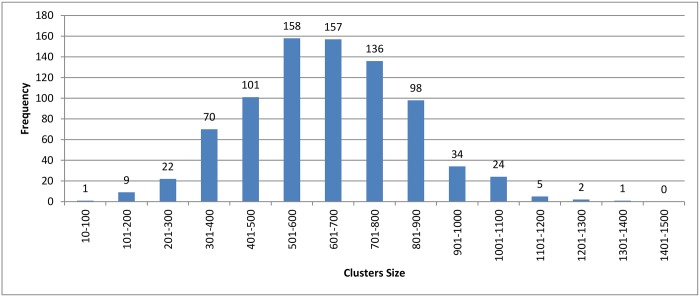
Hierarchical clustering: Clusters distribution for cutoff (*ζ*) = 0.9 with overlapping technique.

**Fig 8:**
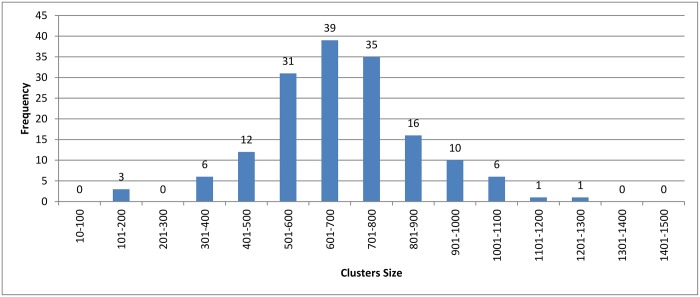
*k*-means clustering: Clusters distribution for *k* = 160 with overlapping technique.

**Fig 9:**
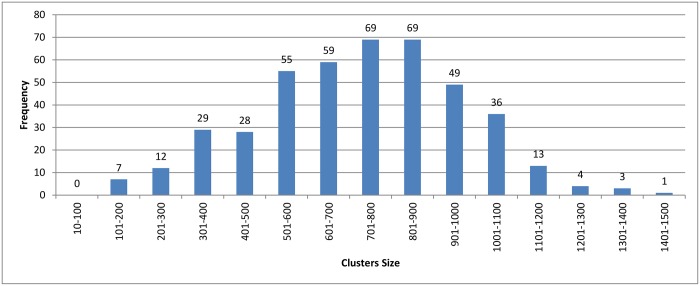
Connected Component clustering: Clusters distribution of threshold (*ψ*) = 95% with overlapping technique.

Figs 7–9 show how the clusters were distributed after applying overlapping. The figures show that the overlapping has led to a normal distribution where we have small frequencies when considering the very large and the very small clusters compared with the other clusters in the same experiment.

**Fig 10:**
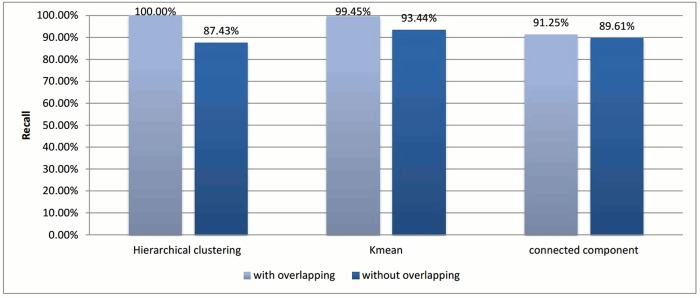
Recall value comparisons for different clustering algorithms using the overlapping technique.

**Fig 11:**
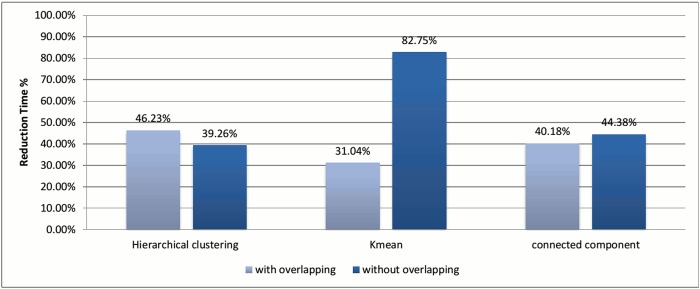
Reduction in time comparison for different clustering algorithms using the overlapping technique.

Figs 10 and 11 illustrate the score measures after applying overlapping to all selected clustering algorithms. Again, this technique is tested with the best experiments for each clustering algorithm, which are used in Section 3.2.

Fig 10 shows that all clustering algorithms achieve better recall after applying the overlapping. It is worth noting that the hierarchical clustering algorithm was enhanced by 12.5% degrees and achieves 100% recall for the input test data, while *k*-means achieved 99.45% recall with an enhancement of approximately 6%. Lastly, connected component achieved an additional 2% enhancement compared to the crisp clustering technique.

In Fig 11, we can see that the hierarchical and the connected component algorithms with overlapping has little impact on the results in terms of reduction in time. In the case of the *k*-means algorithm, the reduction in time is better without overlapping to high extent. This major change in reduction in time can be explained by comparing Figs 8 and 5. In Fig 5, *k*-mean has clustered the profiles without creating any singletons. However, in Fig 8, where overlapping is introduced to *k*-means, the size of clusters has increased in general due to having the same profiles assigned to many clusters. This caused an overhead in the computation time.

### 3.4 Extended evaluation with larger data-set

The previous experiments were conducted for tuning the system parameters. In this section, we carried out a set of experiments with the best-obtained parameters. A larger dataset of 6000 UniRef50 sequence was involved. This dataset was selected randomly from all the UniRef50 data set. We study the recall and reduction in time under different query sizes ranging from 1000 to 6000 sequences.

Table 2 shows the recall for the three different clustering algorithms using different query sizes. We can see that hierarchical clustering has the best average recall value among the three clustering algorithms. Since a larger dataset is involved here, the results are more reliable than the results in the tuning phase. Still, each algorithm occupied its relatively recall order among the others.

**Table 2:** Recall values for different test batch sizes.

Query size	Hierarchical	*k*-means	Connected Component
1000	98.94%	96.34%	97.08%
2000	98.04%	94.96%	95.33%
3000	98.16%	96.11%	91.70%
4000	99.03%	96.93%	94.82%
5000	99.01%	96.91%	91.98%
6000	98.82%	96.75%	92.09%
**average**	98.67%	96.33%	93.83%

Table 3 shows the reduction in time for each algorithm. A higher variety of sequences with widely different sequence lengths were involved in the experiment. When comparing the reduction in time among the three clustering approach, we can see that the values are very close to each others. This is because the three clustering approaches have highly similar cluster distribution after applying the overlapping to them as seen in Figs 7–9.

**Table 3:** Reduction in time for different test batch sizes.

Query size	Hierarchical	*k*-means	Connected Component
1000	41.16%	39.56%	41.38%
2000	41.06%	40.46%	42.33%
3000	40.48%	40.10%	41.96%
4000	41.04%	41.13%	42.32%
5000	40.97%	40.92%	42.11%
6000	40.70%	40.59%	41.75%
**average**	40.90%	40.46%	41.98%

### 3.5 Time Reduction

The proposed approach aims to minimize the retrieval time in profile-profile approach. An experiment to handle a batch query of 1000 sequences took 15.3 hours using the sequential search while it required 9.13 hours with our proposed approach.

## 4 Conclusion

This work proposed a novel information retrieval approach to accelerate the search in profile-HMM databases for homology detection. Unlike the existing techniques, we shift our focus from improving the searching algorithm to reducing the searching scope by representing the database in the form of clusters. From the many existing clustering approaches, we selected three commonly used ones in order to prove the main concept behind this study. These approaches are: *k*-means [29], hierarchical [30], and connected component [31].

These approaches are known as crisp clustering approaches [32]. This means that each profile in the dataset is assigned to only one cluster. To enhance the retrieval process, we introduced an extension step to the clustering process that allows overlapping among the clusters. The overlapping step has enhanced the recall of the system. This was clear in the hierarchical clustering algorithm which produced the best recall over the two other approaches.

When applying connected component and hierarchical clustering, we noticed that many profiles are singletons. which increased the search time. This paper introduced an overlapping technique to solve the search overhead caused by the singletons and to enhance the recall in data retrieval. It is important to emphasize that overlapping among clusters should not be taken into consideration when studying the biological similarities among profiles.

It is important to point that the proposed technique is highly expandable when new profile-HMMs are included in the database. This can be performed by applying the overlapping step to each new profile, and update the related clusters without the need to re-cluster the whole database. Finally, the proposed system can be used to accelerate any other alignment tool other than HHsearch.

## 5 Experimental Details

### 5.1 Setup Time

The establishment of the matrix *S* as well as creating the database of clusters were performed in a multithread manner (32 threads) to obtain it at high speed, while the other steps were carried out using a single core computer in order to study the performance of the proposed approach when running it on low computational power server.

Table 4 demonstrate the time for setting up each testing experiment in section 3.4. This table covers three steps which are: clustering the profiles, applying the overlapping technique and building the clusters databases. In building the clusters database, the listed time is the overall turnaround time not the summation of thread’s time.

**Table 4:** Execution time in minutes for setting up the testing framework.

	Clustring	Overlapping	Building DB
**Connected Component**	0.50	224.70	235.75
**Hierarchical**	2.80	539.95	422.66
**k-means**	27.05	84.95	96.00

### 5.2 Parameter Tuning for hierarchical clustering

For hierarchical clustering experiments, we used the agglomerative hierarchical clustering with the *single* – *link* method found in Matlab. We studied different values of *ζ* and select 0.5, 0.9, and 1.1. These values produced the corresponding cluster size 2839, 2131, and 2007, respectively. *ζ* values either under 0.5 or over 1.1 has no major changes on the number of clusters.

**Fig 12:**
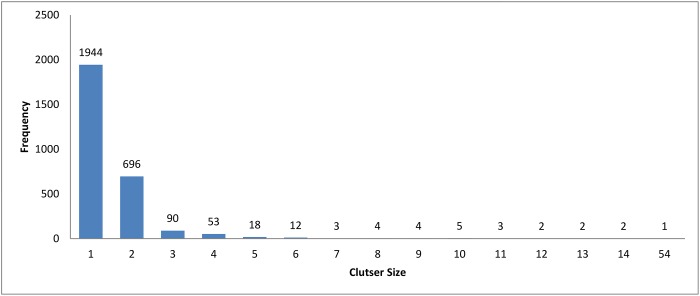
Hierarchical clustering: Clusters distribution for cutoff (*ζ*) = 0.5.

**Fig 13:**
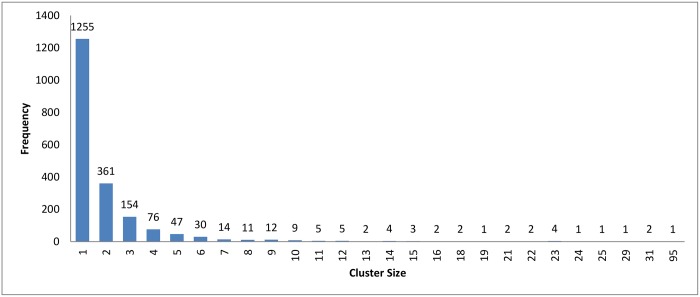
Hierarchical clustering: Clusters distribution for cutoff (*ζ*) = 1.1.

The resulting clusters which are taken from the leaf nodes of the hierarchy based on the cutoff value, are distributed as in Figs 12, 4 and 13. The x-axis displays the size of clusters that are produced by this clustering algorithm, while the y-axis shows clusters frequency for each size. In Fig 12, this algorithm produced 1944 clusters that hold only one profile, while 18 clusters hold 5 profiles and so on. From these figures, we can see that the case of singleton clusters appears in this clustering algorithm. When increasing *ζ*, the number of singletons decreases and, consequently, the size of clusters increases.

**Fig 14:**
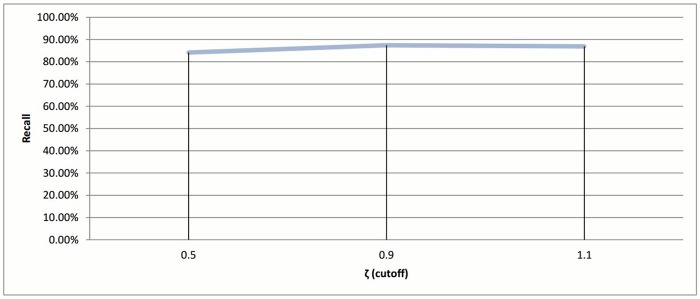
Hierarchical clustering: Recall value measure for different cutoff values (*ζ*).

**Fig 15:**
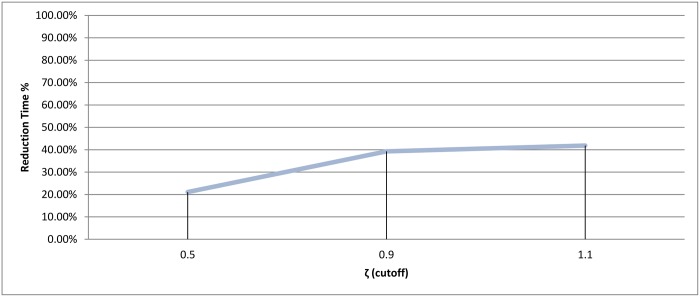
Hierarchical clustering: Reduction time measure for different cutoff values (*ζ*).

If we ignored the singletons, we would have 895, 818, and 752 clusters as a final output for each value 0.5, 0.9, and 1.1, respectively. After preparing all the clusters, the methods for choosing the representatives using a heuristic function were used for testing the technique. The measurement scores for different *ζ* values appear in Figs 14 and 15. The measures show that a medium value of *ζ* gives the best recall value of 87.43%, while the reduction time is 39.26%. The best reduction time obtained by minimum *ζ* with a score value of 41.85% and recall value of 86.88%.

### 5.3 Parameter Tuning for *k*-means clustering experiments

A set of experiments was performed to tune the *k* values (the number of clusters). The squared Euclidean distance was used to calculate the centroid for each cluster. Each centroid cannot be considered as a new profile since the values of this centroid represent average values of the features of the similarity matrix.

k-*means* algorithm initializes the centroid seeds randomly and this can lead to poor clustering. One way to avoid this problem is to have the clustering process repeated many times and obtain the best clustering pattern. However, to avoid repeating the clustering many times, we relied on k-means++ algorithm [33] under MatLab. This algorithm uses a heuristic process for spreading out the initial centroid seeds.

**Fig 16:**
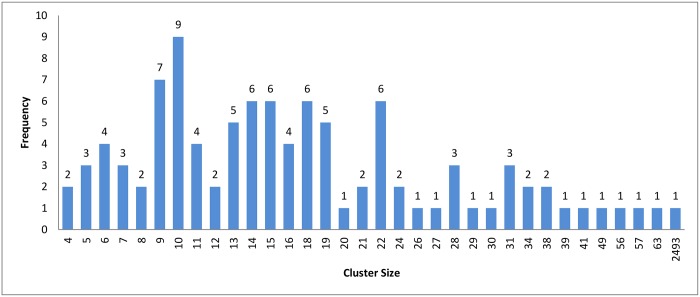
*k*-means clustering: Cluster distribution for *k* = 100.

**Fig 17:**
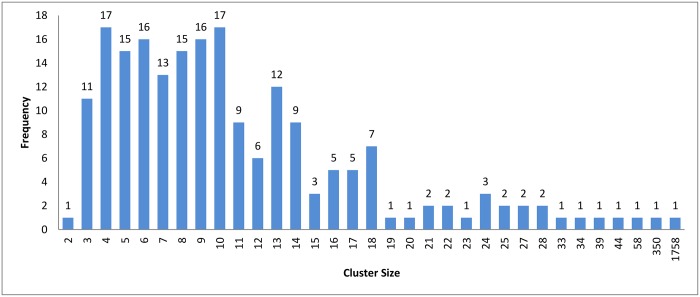
*k*-means clustering: Cluster distribution for *k* = 200.

**Fig 18:**
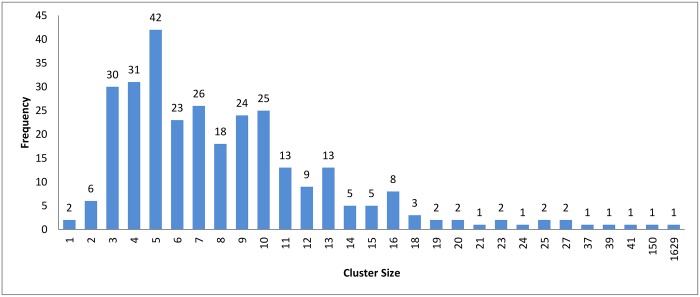
*k*-means clustering: Cluster distribution for *k* = 300.

**Fig 19:**
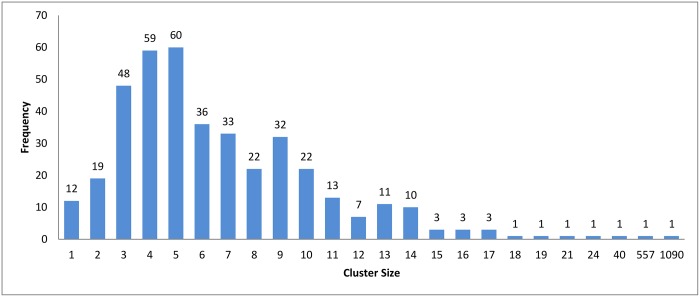
*k*-means clustering: Cluster distribution for *k* = 400.

**Fig 20:**
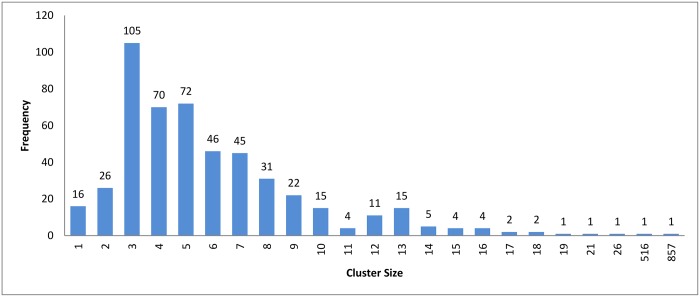
*k*-means clustering: Cluster distribution for *k* = 500.

**Fig 21:**
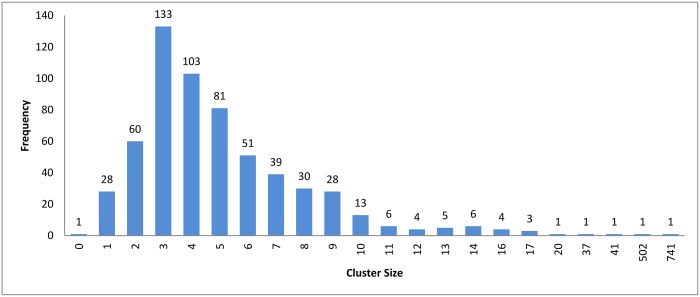
*k*-means clustering: Cluster distribution for *k* = 600.

We carried out a set of experiments with different values of *k* equal to [100, 200, 300, 400, 500, 600]. The cluster distribution for these experiments is shown in Figs 16–21 respectively. From these experiments, singletons disappeared when *k* equals 100 and 200, while a very little number of them exists when *k* is greater than or equals 300.

**Fig 22:**
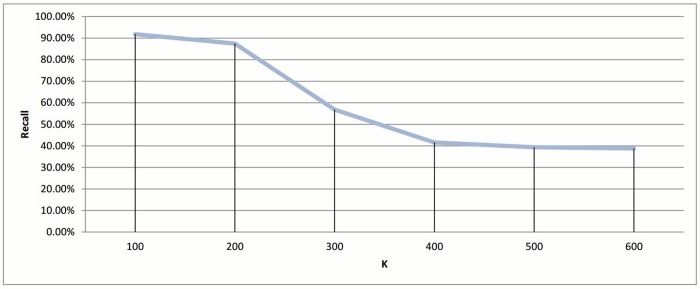
*k*-means clustering: Recall value measure for different *k* value.

**Fig 23:**
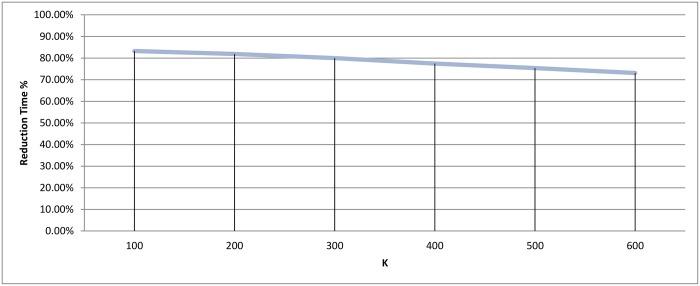
*k*-means clustering: Reduction time measure for different *k* values.

When the number of clusters increases, the number of small clusters also increases, bearing in mind that there is always one relatively large cluster. The size of clusters is inversely proportional to the number of clusters. Finally, we stopped the experiments when we reach *k* = 600 because empty clusters start to appear as seen in Fig 21. After preparing all the clusters, the representatives were chosen based on the *heuristic-representative* approach. The recall and reduction in time of these six experiments are displayed in Figs 22 and 23.

The best scores were achieved when *k* = 100, with recall value being 91.80%, and the reduction in time being 83.25%. As the number of clusters increased, both recall and reduction in time were negatively affected. It is logical for there to be a decline in the reduction of time measurement as the number of clusters increases, since this increase also causes an increase in the number of representatives, which would necessitate more comparisons for each query. In addition, having more clusters usually leads to higher possibility for the query to miss the target cluster i.e. (decline in the recall value)

**Fig 24:**
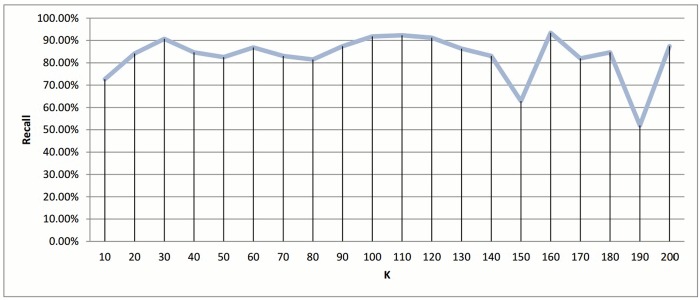
*k*-means clustering: Recall value measure for different *k* value.

**Fig 25:**
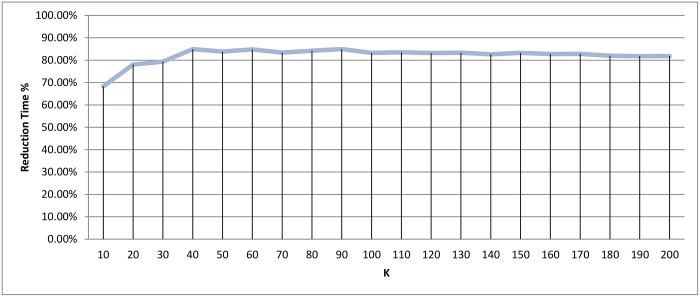
*k*-means clustering: Reduction time measure for different *k* values.

Since the best results were achieved when the value of *k* is at the boundary, another set of experiments was run to cover a closer interval for the best peak point. These results appear in Figs 24 and 25, and shows that the *k* = 100 region was considered a good local maximum interval, with the best recall value in that region being when *k* = 110 with a score of 92.34%. Out of all *k* values, the best recall value was achieved when *k* = 160 with a score of 93.44%, and a reduction in time of 83.53% and 82.75% for *k* equals 110 and 160, respectively.

Another point worth noting is that as *K* decreases the reduction in time measurement is negatively affected. The number of representatives is small, which requires a fewer number of comparisons to be made between the query and representatives. However, the size of the clusters increases, which increases the number of comparisons required for each query. A cluster distribution when *k* = 160 is displayed in Fig 5.

### 5.4 Parameter Tuning for connected component clustering

In connected component clustering algorithm, the profiles are represented as vertices in an undirected graph, while the similarity between any two profiles in this graph is presented as a weighted edge from *S*. Here, clustering is done by dividing the graph to sub-graphs by removing all the edges that have a weight less than a given threshold *ψ*. In this section, we applied connected component clustering. Three values for *ψ* were used as a threshold; these values are 99%, 95% and 90%. The reason for using these three values is to observe how the technique will behave when a threshold goes from the maximum value possible to a value that is lower than the default similarity score which is 95% (see Section 2.3).

**Fig 26:**
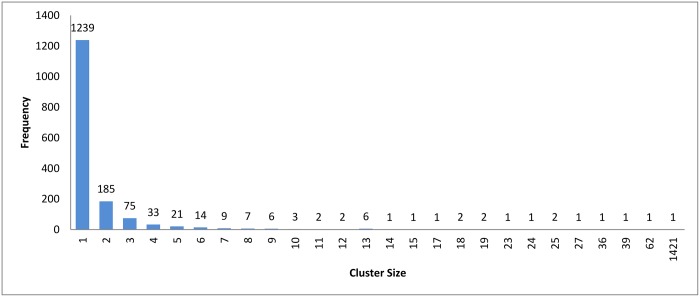
Connected component clustering: Clusters distribution for threshold (*ψ*) = 90%.

**Fig 27:**
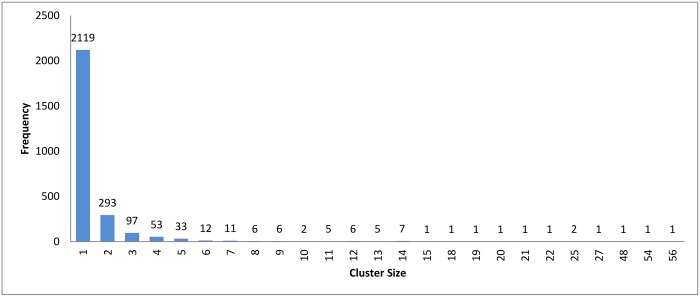
Connected component clustering: Clusters distribution for threshold (*ψ*) = 99%.

The cluster distribution for these experiments is shown in Figs 26, 6 and 27 respectively. From these experiments, The decrease in threshold will leads to decrease the number of clusters, a number of singletons and increase the clusters size. This is logic since relaxing threshold value means fewer edges need to be removed.

When ignoring singletons, the results become as follows: 548 clusters when *ψ* = 99%, 434 clusters when *ψ* = 95% and finally 379 clusters when *ψ* = 90% as a final output.

**Fig 28:**
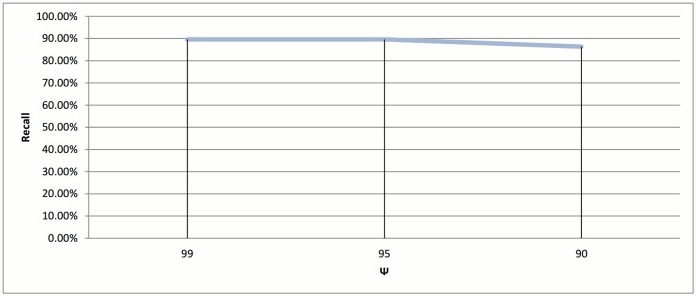
Connected component clustering: Recall value measure for different thresholds (*ψ*).

**Fig 29:**
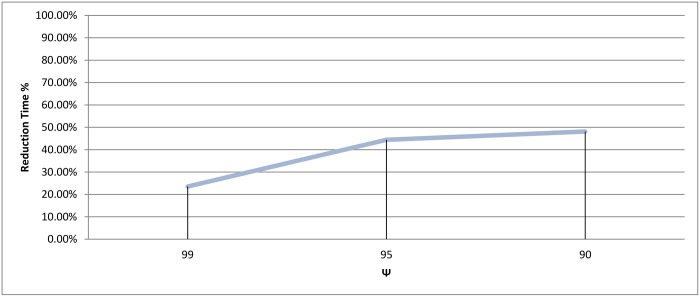
Connected Component clustering: Reduction time measure for different thresholds (*ψ*).

The score measurements for all *ψ* testes values are shown in Figs 28 and 29. The best recall value appears when *ψ* equal to 95% and 99% with value 89.61%, while the reduction time scores better when the threshold is 95% with value 44.38%.

## Supporting Information

S1 File Support materials for executing the experiments. A compressed (.zip) file that contains all the needed scripts for executing any experiment of this work is provided. The similarity matrix is also included. The README file contains the needed details to run these scripts.
(ZIP)
