# Supplementary material for: Accelerating Information Retrieval from Profile Hidden Markov Model Databases
Source: PLoS One. 2016 Nov 22;11(11):e0166358. doi: 10.1371/journal.pone.0166358 (PMC5119741; doi:10.1371/journal.pone.0166358)
Supplement: S1 File — A compressed (.zip) file that contains all the needed scripts for executing any experiment of this work is provided. The similarity matrix is also included. The README file contains the needed details to run these scripts. (ZIP) [file pone.0166358.s001.zip › supported material/read me.txt.docx]

To set up the experiment on Linux OS please make sure to do the following steps:

1- Download the seeds from the 13th release of TIGFAM database

2- Install and configure hhsuite-2.0.16 package on your device.

3- Build the following directory structure :

/exp : The root directory

/exp/data : The root directory for experiment data

/exp/data/tf13_seeds : Will hold the TIGRFAMs profiles seeds

/exp/data/tf13_a3m : Will hold the formatted seeds (fasts -> a3m)

/exp/data/tf13_a3m_updated : Will hold the a3m files after renaming the sequences in all files with a sequential integer number

/exp/data/tf13_hhm : Will hold the generated profile-hmm using hhsearch suit for getting more accurate scores

/exp/data/tf13_hhm_db : Will hold the database for the hhm collection to be used later in any search and scoring process

/exp/out : Will hold the output result from the comparison process

/exp/formatedRes : A temporary directory for holding the hhsearch profile-profile scores after extract them from the original score file.

/exp/finalResults : Will hold the final result from the base process, like the similarity matrix with different scoring type, name mapping

for each profile, and similarity matrix index file.

/exp/runRes/ : Will hold the representatives, representatives database, and the database for each cluster. Many directories will auto generated

inside it, and many other inside each one.

4- Extract the downloaded seeds in point 1 to the directory /exp/data/tf13_seeds

5- Run the Matlab script step_1.m, which used to change the Fasta seeds files to a3m format and move the results to /exp/data/tf13_a3m,

then rename each sequence header with a sequential number. This step important because HHsearch used the first sequence head in each file as a profile

name, and since Matlab has a limit charachter number in string comparison. Leaving the head as is will cause a problem later in the comparison process.

As a workaround solution, this step comes to index the profiles for smooth comparison later on. An index file is created so you can get back the sequences

original headers.

6- Run the shell script step_2.sh, which will

- Create all the HMM profiles and save them to /exp/data/tf13_hhm

- Build the based profile database and save it to /exp/data/tf13_hhm_db

- Do all the needed comparisons for building the all-against-all similarity matrix and put the results in /exp/formatedRes.

7- Run the Matlab scritp step_3.m, which will

- Generate a details text file for mapping between our indexing profile name and its actual name and save the result to /exp/finalResults/det.txt.

- Generate the similarity matrix based on the probability measure from HHsearch tool results, and save the result to /exp/finalResults/prob.txt.

- Generate an index file for sorting the values in the similarity matrix, and save it to /exp/finalResults/resultMatIndex.txt. This step is important

since the compared results sorted by default based on the similarity, so this sort index needed to put each value in its correct location wherever it

appears in the compared result file.

8- To run k-means, connected component and hierarchical algorithms run the following scripts:

- k_mean.m

- connected_component.m

- hc.m

Each on of those scripts will do the following:

- Clustering the data.

- Select the representatives.

- Build a database for each cluster.

- Build a database for the representatives.

- Apply overlapping.

- Testing.

General Notes:

- Use one of the virtual process managers, like (screen). This needed if you working via ssh, since some part of this script may take many hours.

- det.txt, prob.txt, resultMatIndex.txt files are included with this material.

- Multiple sequence alignment does not exist in those scripts. However, I used the Matlab command "multialign" to generate the MSA-Representative.

Tow important issues with multialign:

+ It is not working with less than 3 sequnces in the aligned file. So if the file contains one sequence repeate it twice and if the file

contains 2 sequences double them once.

+ Don't forget to remove U and X from sequences, since matlab function will raise an error if u leave them.

- When downloading sequences from uniref, you will get them all in one fasta file. So I split each sequence in a file using splitFasta.cpp code.

Then select a random set of them for testing.

- Warning: some variables are manually assigned in the scripts or manually re-assigned.
